# Supplementary material for: HIFα isoform specific activities drive cell-type specificity of VHL-associated oncogenesis
Source: Nat Commun. 2025 Oct 16;16:9185. doi: 10.1038/s41467-025-64214-3 (PMC12533196; doi:10.1038/s41467-025-64214-3)
Supplement: Supplementary file 1 — Supplementary Information File [file 41467_2025_64214_MOESM1_ESM.pdf]

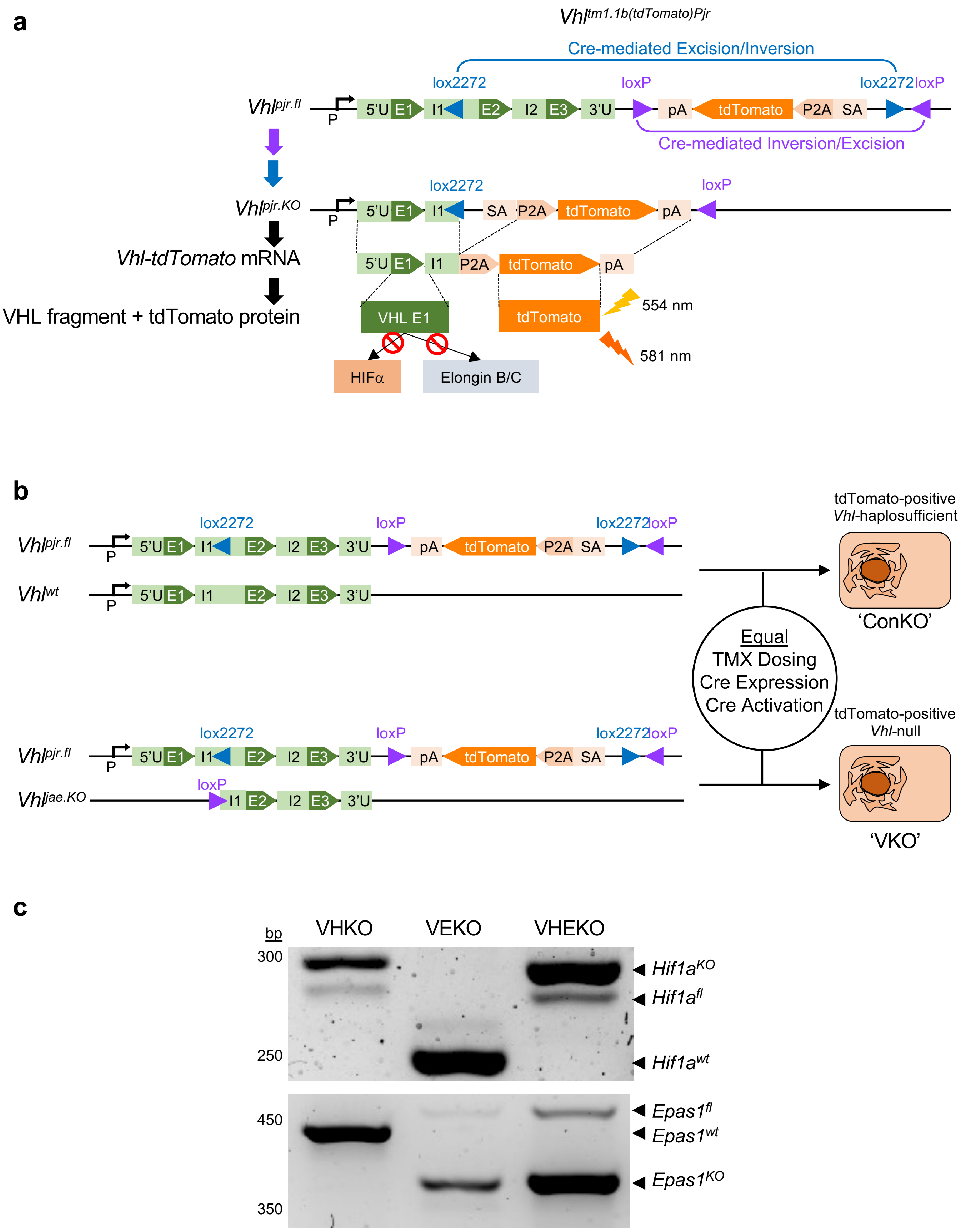
a
Vhl tm1.1b(tdTomato)Pjr
Cre-mediated Excision/Inversion
lox2272
loxP
lox2272
loxP
Vhl pjr.fl
P
5'U E1 I1 E2 I2 E3 3'U
pA tdTomato P2A SA
Cre-mediated Inversion/Excision
Vhl pjr.KO
5'U E1 I1 SA P2A tdTomato pA
Vhl-tdTomato mRNA
5'U E1 I1 P2A tdTomato pA
VHL fragment + tdTomato protein
VHL E1
tdTomato
554 nm
581 nm
HIFα
Elongin B/C
b
tdTomato-positive
Vhl-haplosufficient
Vhl wt
'ConKO'
Equal
TMX Dosing
Cre Expression
Cre Activation
tdTomato-positive
Vhl-null
Vhl jae.KO
'VKO'
c
bp
VHKO
VEKO
VHEKO
300
250
450
350
Hif1a KO
Hif1a fl
Hif1a wt
Epas1 fl
Epas1 wt
Epas1 KO

Supplementary Fig. 1: Co-deletion of *Hif1a* and *Epas1* in tdTomato-tagged *Vhl*-recombined cells.

(a) Schematic diagram depicting the design and recombination of the $Vhl^{pjr.fl}$ allele. $Vhl^{pjr.fl}$ and $Vhl^{pjr.KO}$ refer to 'floxed' and 'knockout' forms of the Vhl^{pjr} allele. P, *Vhl* promoter; U, untranslated region; E, *Vhl* exon; I, *Vhl* intron; pA, polyadenylation site; P2A, porcine teschovirus 2A peptide; SA, splice acceptor. Dashed lines - spliced and translated regions; lightning symbols - excitation and emission wavelengths for tdTomato fluorescence. Red crossed circle indicates no interaction between VHL exon 1 fragment and HIFα or Elongin B/C. **(b)** *Vhl* allele construction for ConKO and VKO mice. Mice of both genotypes carry the $Vhl^{pjr.fl}$ allele. ConKO mice carry a second wild-type Vhl^{wt} allele, while VKO mice carry a constitutively inactivated $Vhl^{jae.KO}$ allele. **(c)** Genomic PCR for *Hif1a* and *Epas1* alleles performed on FAC-sorted tdTomato-positive cells from VHKO, VEKO, and VHEKO mice given 5x 2 mg tamoxifen and harvested early (1-3 weeks) after recombination. Expected amplicon sizes for wild-type (wt), floxed/unrecombined (fl) and knock-out/recombined (KO) forms of *Hif1a* and *Epas1* depicted by black arrows.

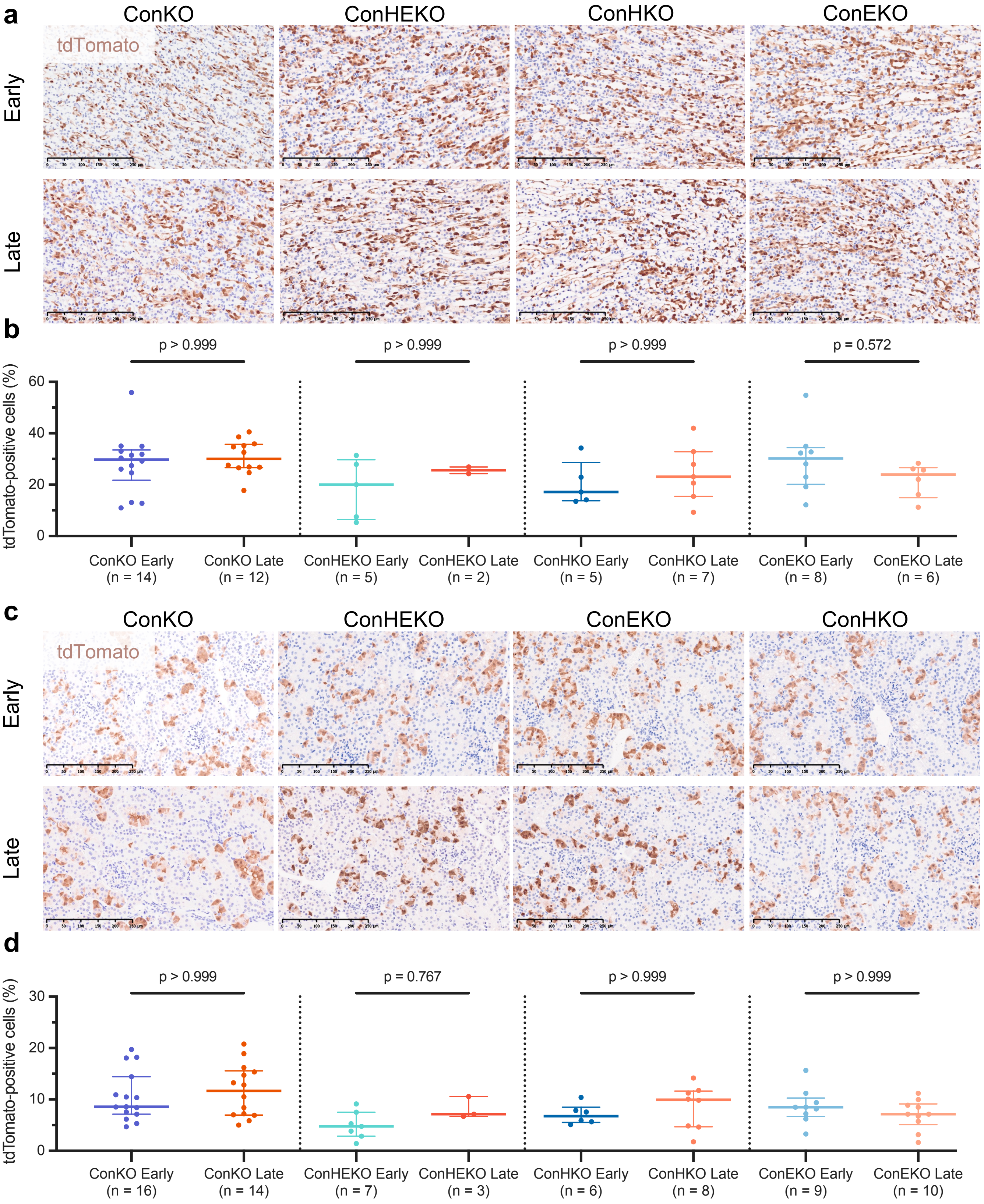
a
ConKO
ConHEKO
ConHKO
ConEKO
tdTomato
Early
Late
b
p > 0.999
p > 0.999
p > 0.999
p = 0.572
tdTomato-positive cells (%)
0
20
40
60
ConKO Early (n = 14)
ConKO Late (n = 12)
ConHEKO Early (n = 5)
ConHEKO Late (n = 2)
ConHKO Early (n = 5)
ConHKO Late (n = 7)
ConEKO Early (n = 8)
ConEKO Late (n = 6)
c
ConKO
ConHEKO
ConEKO
ConHKO
tdTomato
Early
Late
d
p > 0.999
p = 0.767
p > 0.999
p > 0.999
tdTomato-positive cells (%)
0
10
20
30
ConKO Early (n = 16)
ConKO Late (n = 14)
ConHEKO Early (n = 7)
ConHEKO Late (n = 3)
ConHKO Early (n = 6)
ConHKO Late (n = 8)
ConEKO Early (n = 9)
ConEKO Late (n = 10)

Supplementary Fig. 2: Response to HIFα inactivation in different renal regions of 'control' kidneys.

(a, c) Representative tdTomato IHC counterstained with hematoxylin in the renal papilla **(a)** or renal cortex and outer medulla **(c)** of ConKO, ConHKO, ConEKO, and ConHEKO mice given 5x 2 mg tamoxifen and harvested early (1-3 weeks) or late (4-12 months) following recombination. Scale bar denotes 250 μm; 20x magnification. **(b, d)** Automated quantification (see **Methods**) of the proportion of cells that are tdTomato-positive in the renal papilla **(b)** or renal cortex and outer medulla **(d).** Pairwise comparisons by Kruskal-Wallis test with Dunn's correction. Data are presented as median values, with the inter-quartile range indicated by error bars.

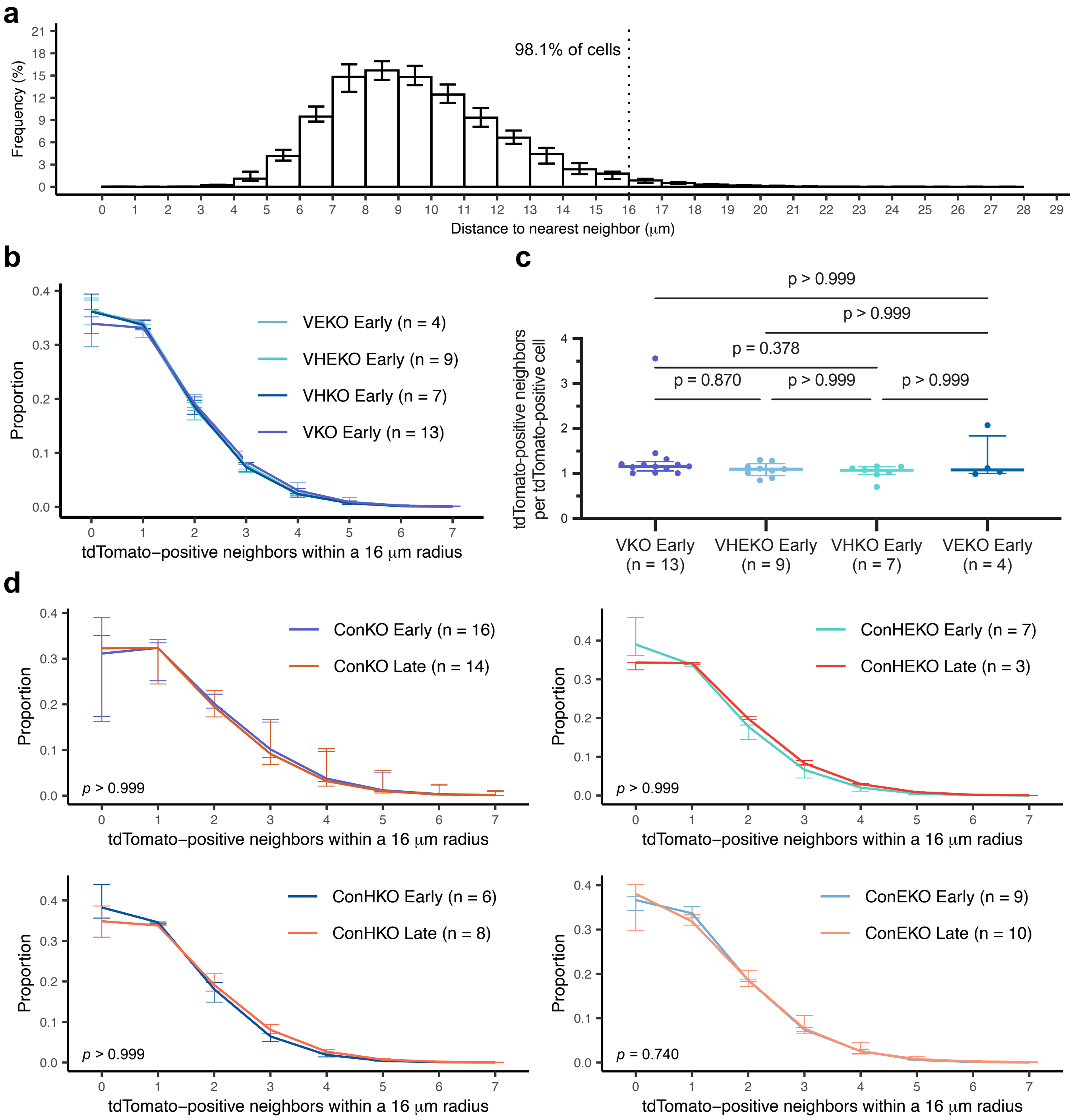

a
98.1% of cells
Frequency (%)
Distance to nearest neighbor (μm)
b
VEKO Early (n = 4)
VHEKO Early (n = 9)
VHKO Early (n = 7)
VKO Early (n = 13)
Proportion
tdTomato−positive neighbors within a 16 μm radius
c
p > 0.999
p > 0.999
p = 0.378
p = 0.870
p > 0.999
p > 0.999
tdTomato-positive neighbors per tdTomato-positive cell
VKO Early (n = 13)
VHEKO Early (n = 9)
VHKO Early (n = 7)
VEKO Early (n = 4)
d
ConKO Early (n = 16)
ConKO Late (n = 14)
p > 0.999
ConHEKO Early (n = 7)
ConHEKO Late (n = 3)
p > 0.999
ConHKO Early (n = 6)
ConHKO Late (n = 8)
p > 0.999
ConEKO Early (n = 9)
ConEKO Late (n = 10)
p = 0.740
Proportion
tdTomato−positive neighbors within a 16 μm radius

Supplementary Fig. 3: Spatial distribution of cells in the renal cortex and outer medulla.

(a) Histogram depicting the distance to the nearest neighbor, whether tagged or untagged, for tdTomato-positive cells in the renal cortex and outer medulla. Median and interquartile range indicated. 50,000 cells analyzed over n = 10 mice chosen randomly across all genotypes and timepoints. **(b, c)** Frequency distribution **(b)**, and mean number **(c)** of tdTomato-positive neighbors of tdTomato-positive cells within a 16 μm radius in the cortex and outer medulla of VKO, VHEKO, VHKO, and VEKO mice harvested early (1-3 weeks) after *Vhl* inactivation. Data are presented as median values, with the inter-quartile range indicated by error bars. Pairwise comparisons by Kruskal-Wallis test with Dunn's correction. **(d)** Frequency distribution of tdTomato-positive neighbors of tdTomato-positive cells within a 16 μm radius in the cortex and outer medulla of ConKO, ConHEKO, ConHKO, and ConEKO mice harvested either early or late after *Vhl* inactivation. Frequency distributions compared using Wilk's lambda statistic in multivariate analysis of variance (MANOVA).

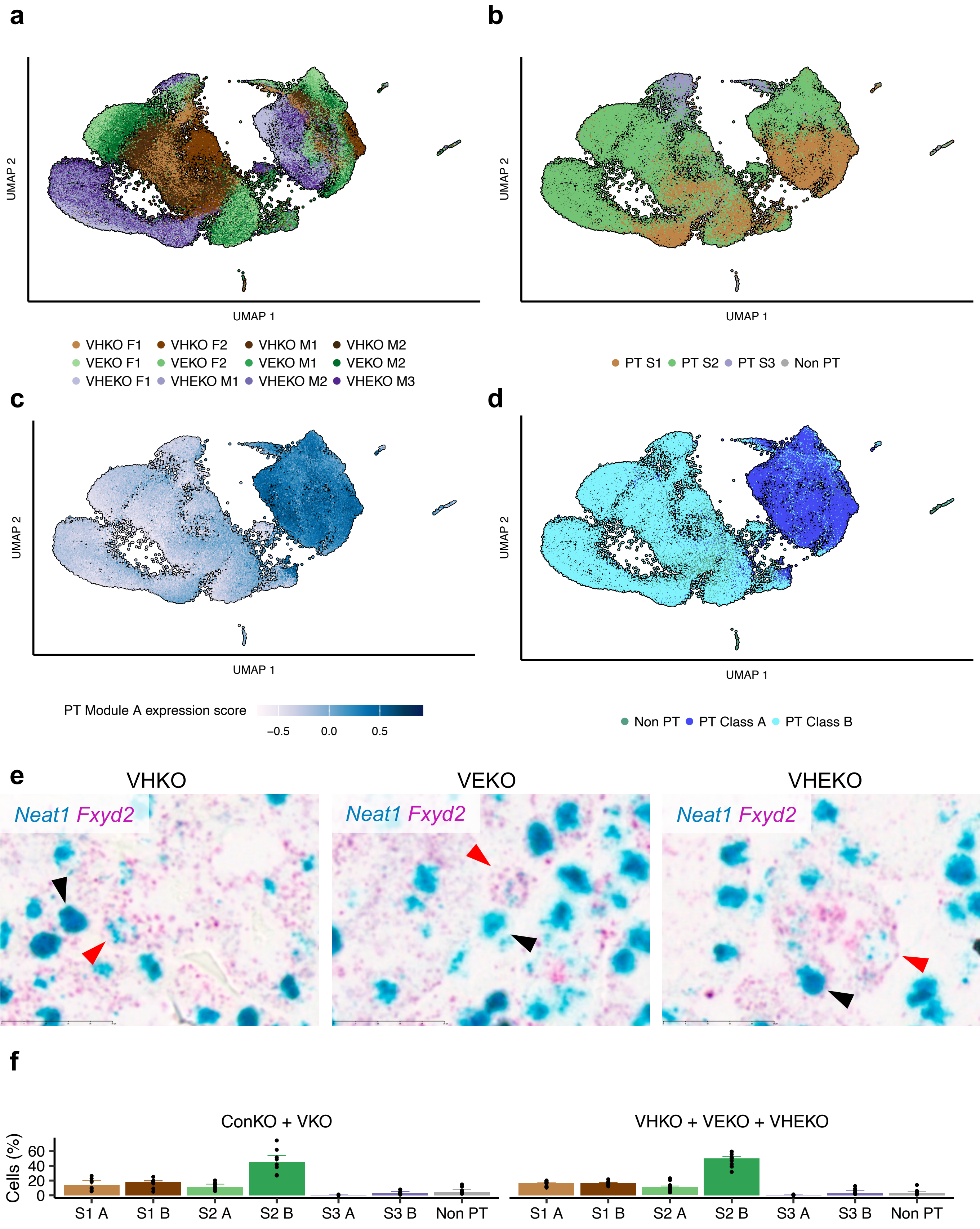

a
b
UMAP 2
UMAP 1
VHKO F1
VHKO F2
VHKO M1
VHKO M2
VEKO F1
VEKO F2
VEKO M1
VEKO M2
VHEKO F1
VHEKO M1
VHEKO M2
VHEKO M3
PT S1
PT S2
PT S3
Non PT
c
d
PT Module A expression score
−0.5
0.0
0.5
Non PT
PT Class A
PT Class B
e
VHKO
VEKO
VHEKO
Neat1 Fxyd2
f
ConKO + VKO
VHKO + VEKO + VHEKO
Cells (%)
60
40
20
0
S1 A
S1 B
S2 A
S2 B
S3 A
S3 B
Non PT

Supplementary Fig. 4: Single-cell RNA sequencing of tdTomato-tagged cells from VHKO, VEKO, and VHEKO mice.

(a-d) UMAP plots depicting tdTomato-positive cells of n = 4 VHKO, VEKO, and VHEKO mice harvested at 4-12 months following *Vhl* and *Hif1a* and/or *Epas1* inactivation. **(a)** Data distribution by individual mouse. Cell colors are shaded according to the individual mouse within the genotypic group (VHKO in shades of brown; VEKO in shades of green; VHEKO in shades of purple). M = Male; F = Female. Concordance of the shades of the colors in UMAP space illustrates the reproducibility of the data. **(b)** Data distribution by assigned renal cell type. Proximal Tubule (PT). Cells of the same cell type are positioned together in UMAP space. **(c)** Expression score for PT Module A. High and low expression of PT Module A associates with distinct areas in UMAP space. **(d)** Data distribution by PT class as defined by PT Module A expression score. **(e)** Representative dual RNA *in situ* hybridization for *Neat1* (blue) and *Fxyd2* (pink) mRNA in kidney sections from VHKO, VEKO, and VHEKO mice. PT Class A ($Neat1^{high}/Fxyd2^{low}$; black arrows) and PT Class B ($Neat1^{low}/Fxyd2^{high}$; red arrows) cells are indicated. **(f)** Proportion of cells (median and inter-quartile range) that are of each PT identity in scRNA-seq datasets of ConKO and VKO (left), or VHKO, VEKO, and VHEKO (right) cells. Both datasets exhibit similar composition of PT identities. **(b, d)** All cell types other than PT S1, S2, and S3 cell types are assigned as 'Non PT'.

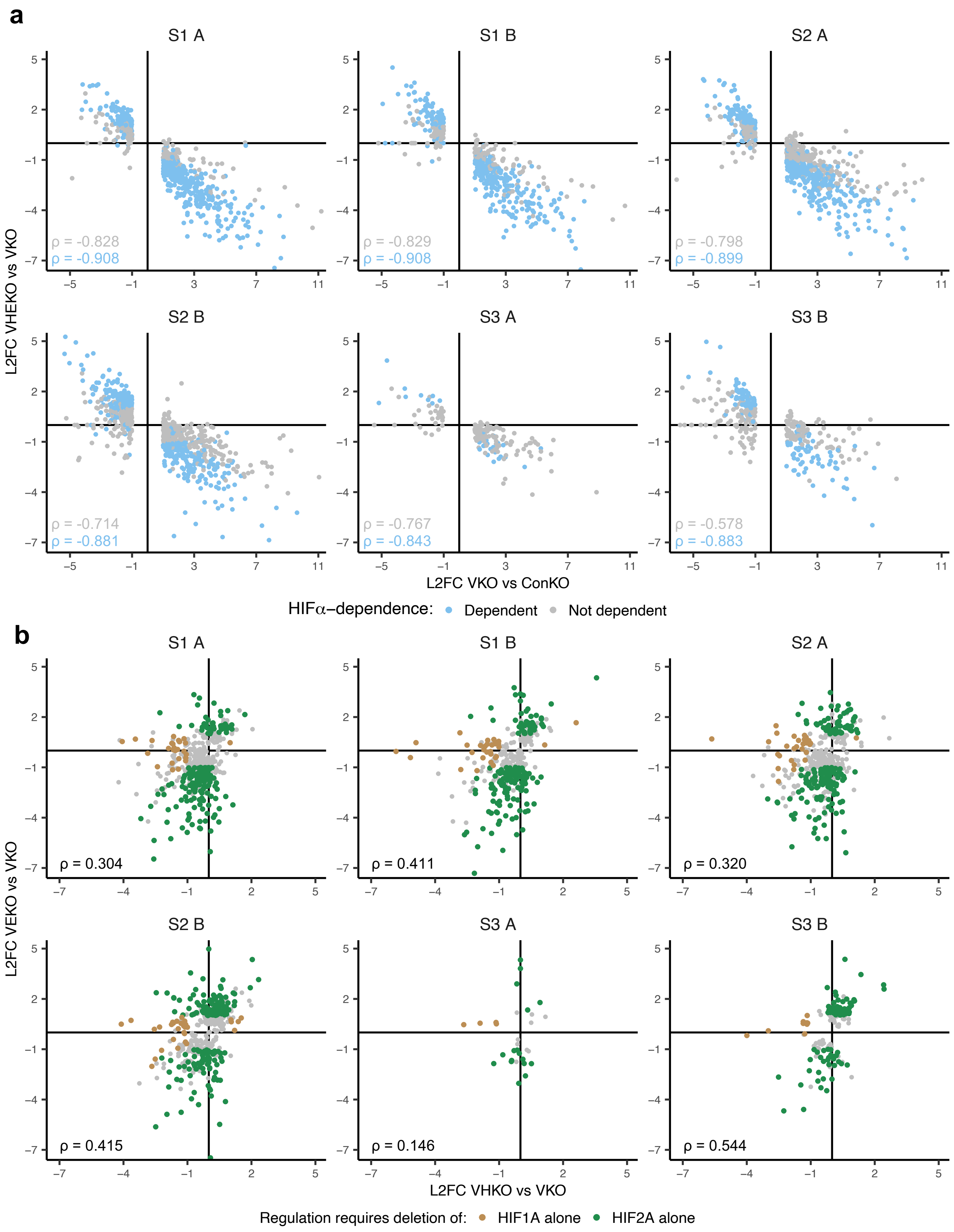

a
S1 A
S1 B
S2 A
S2 B
S3 A
S3 B
ρ = -0.828
ρ = -0.908
ρ = -0.829
ρ = -0.908
ρ = -0.798
ρ = -0.899
ρ = -0.714
ρ = -0.881
ρ = -0.767
ρ = -0.843
ρ = -0.578
ρ = -0.883
L2FC VHEKO vs VKO
L2FC VKO vs ConKO
HIFα–dependence: Dependent Not dependent
b
S1 A
S1 B
S2 A
S2 B
S3 A
S3 B
ρ = 0.304
ρ = 0.411
ρ = 0.320
ρ = 0.415
ρ = 0.146
ρ = 0.544
L2FC VEKO vs VKO
L2FC VHKO vs VKO
Regulation requires deletion of: HIF1A alone HIF2A alone

Supplementary Fig. 5: HIFα isoform-specific dependence of gene expression in *Vhl*-null cells.

(a) Effects of combined *Hif1a* and *Epas1* deletion on expression of *Vhl*-dependent genes in each PT identity. Each scatter plot depicts pseudo-bulked log_2-fold changes (L2FC) for *Vhl*-dependent genes in VHEKO *versus* VKO mice (HIFα-dependence) plotted against the changes in VKO *versus* ConKO mice (*Vhl* dependence) harvested late after recombination. Genes defined as significantly regulated by HIFα co-deletion (blue) and those whose alteration was not significant (grey). Spearman correlation coefficients (ρ) provided separately for genes defined (blue) or not defined (grey) as significantly HIFα-dependent. Note that both significant and non-significant regulation by HIFα is anti-correlated with regulation by *Vhl*. **(b)** Isoform-specific effects of *Hif1a* and *Epas1* deletion on *Vhl*-dependent genes in each PT identity. Each scatter plot depicts pseudo-bulked L2FC for *Vhl*-dependent genes in VHKO *versus* VKO mice (HIF1A-dependence) plotted against the changes in VEKO *versus* VKO mice (HIF2A-dependence) harvested late after recombination. Spearman correlation coefficient (ρ). Note that regulation by HIF1A is not strongly correlated with regulation by HIF2A. Genes whose regulation was reversed significantly by individual deletion of *Hif1a* but not *Epas1* (‘HIF1A alone’; brown) and by *Epas1* but not *Hif1a* (‘HIF2A alone’; green). Other HIFα-dependent genes (grey).

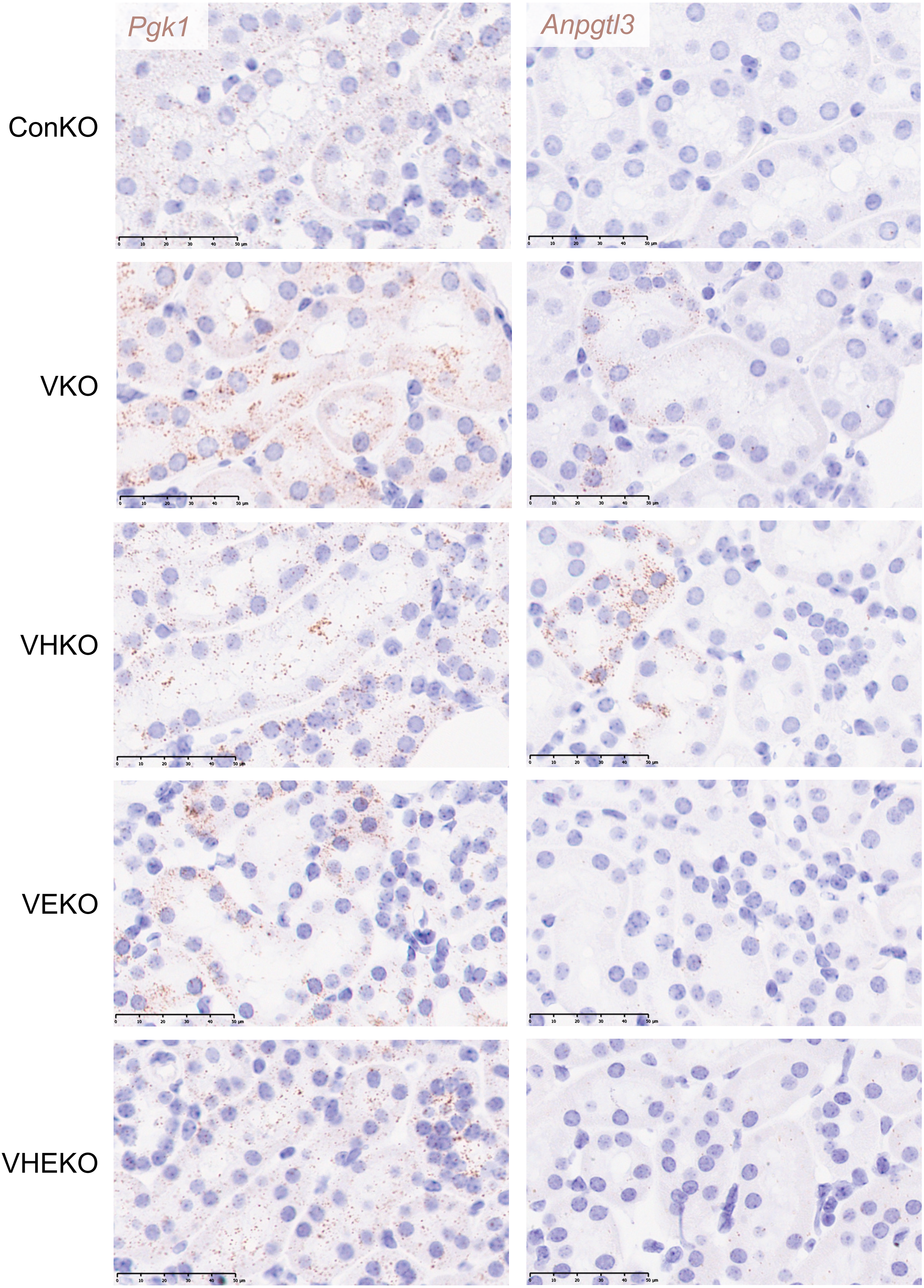
Pgk1
Anpgtl3
ConKO
VKO
VHKO
VEKO
VHEKO

Supplementary Fig. 6: HIFα isoform-specific dependent regulation of *Vhl*-dependent genes.

Representative RNA *in situ* hybridization depicting the expression of HIF1A-specific *Pgk1* (left) and HIF2A-specific *Angptl3* (right) in renal cortex of ConKO, VKO, VHKO, VEKO, and VHEKO mice given 5x 2 mg tamoxifen and harvested late (4-12 months) following recombination. Scale bar denotes 50 μm; 40x magnification.

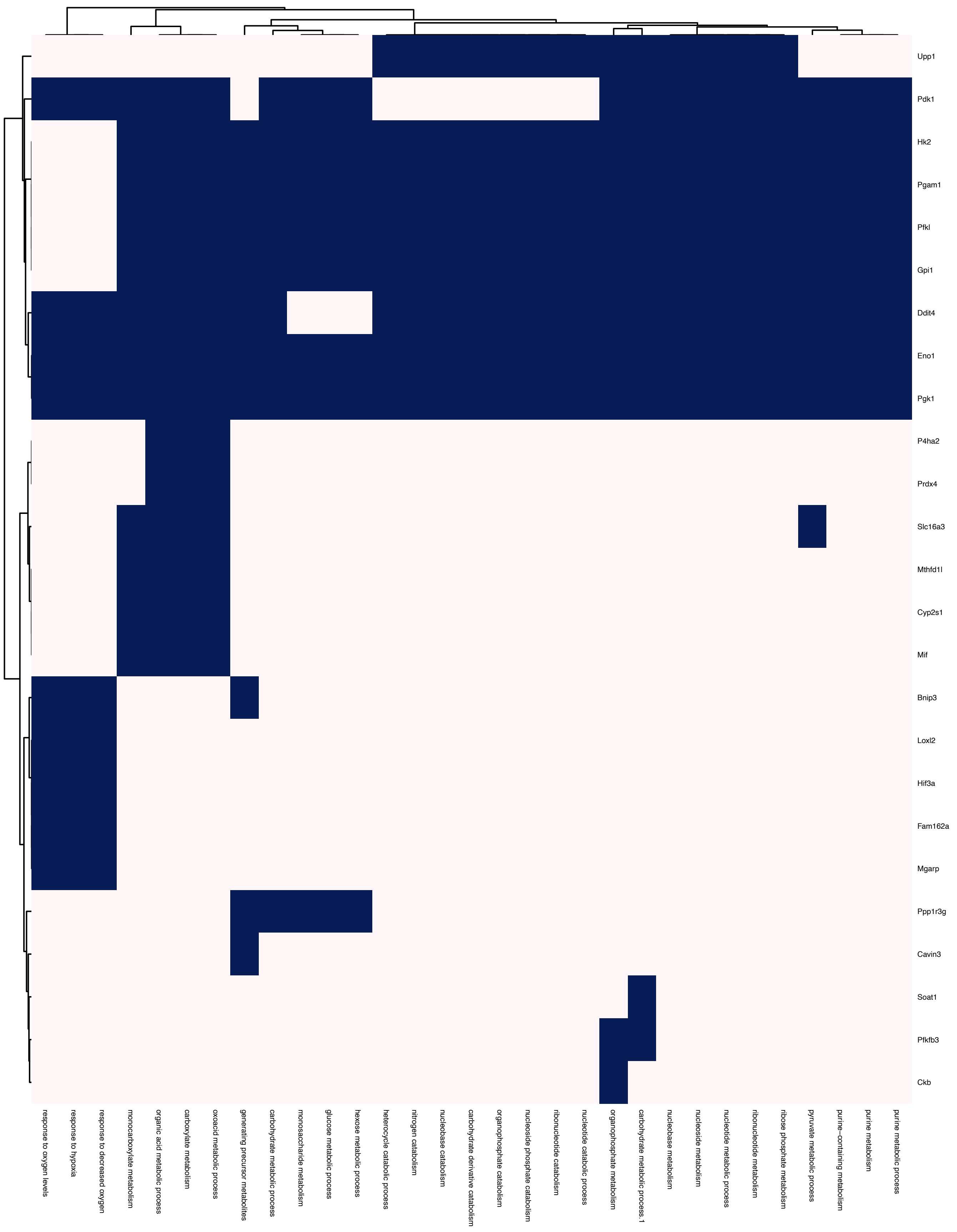

Upp1
Pdk1
Hk2
Pgam1
Pfkl
Gpi1
Ddit4
Eno1
Pgk1
P4ha2
Prdx4
Slc16a3
Mthfd1l
Cyp2s1
Mif
Bnip3
Loxl2
Hif3a
Fam162a
Mgarp
Ppp1r3g
Cavin3
Soat1
Pfkfb3
Ckb
response to oxygen levels
response to hypoxia
response to decreased oxygen
monocarboxylate metabolism
organic acid metabolic process
carboxylate metabolism
oxoacid metabolic process
generating precursor metabolites
carbohydrate metabolic process
monosaccharide metabolism
glucose metabolic process
hexose metabolic process
heterocycle catabolic process
nitrogen catabolism
nucleobase catabolism
carbohydrate derivative catabolism
organophosphate catabolism
nucleoside phosphate catabolism
ribonucleotide catabolism
nucleotide catabolic process
organophosphate metabolism
carbohydrate metabolic process.1
nucleobase metabolism
nucleoside metabolism
nucleotide metabolic process
ribonucleotide metabolism
ribose phosphate metabolism
pyruvate metabolic process
purine−containing metabolism
purine metabolism
purine metabolic process

Supplementary Fig. 7: **Individual HIF1A-specific upregulated genes driving over-representation in the GO terms.**

Binary heatmap depicting whether individual HIF1A-specific upregulated genes are or are not members of the indicated GO terms. Blue colored tiles represent membership of the gene in the GO term. GO terms and genes are ordered by hierarchical clustering based on the overlapping memberships of GO terms.

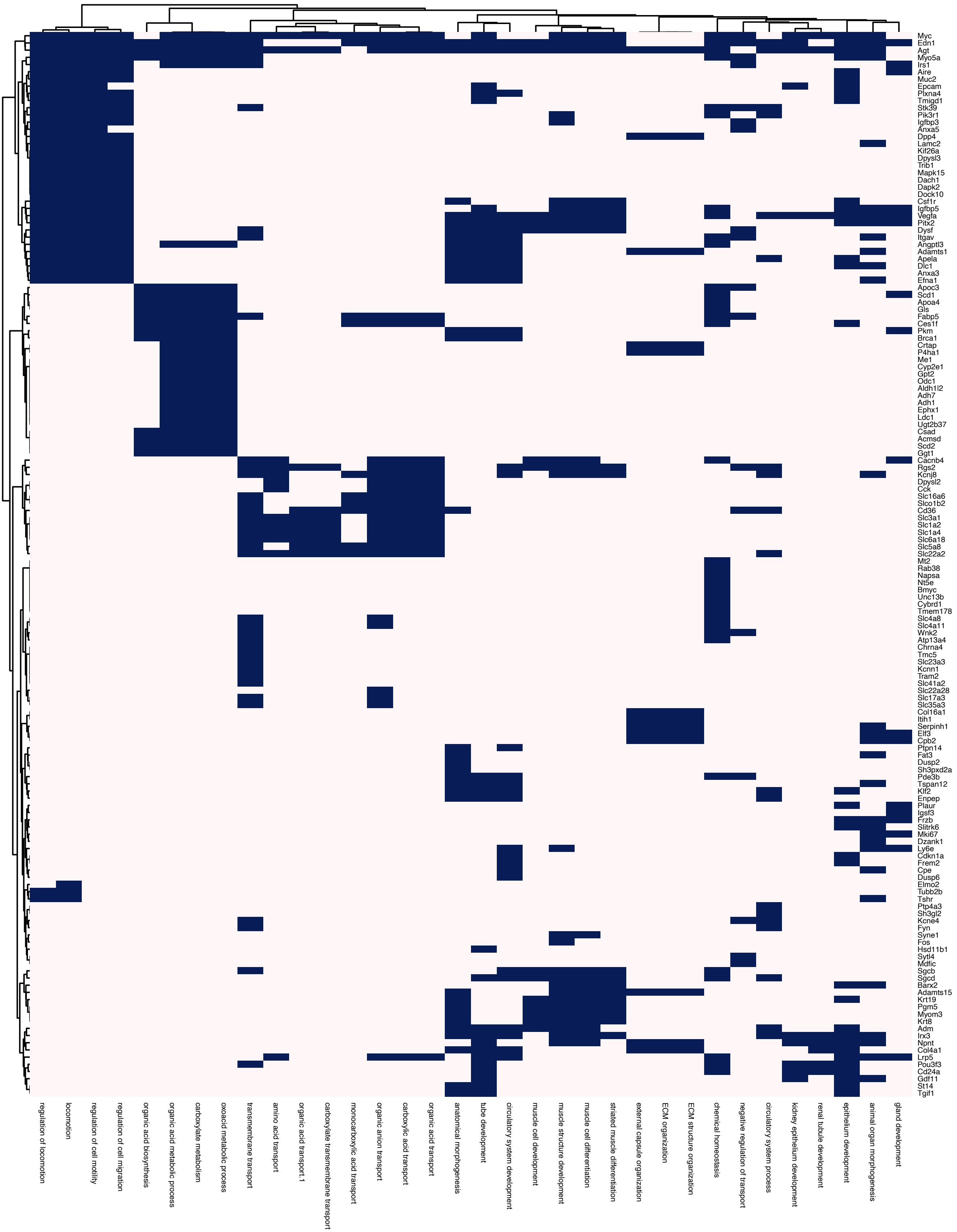
Myc
Edn1
Agt
Myo5a
Irs1
Aire
Muc2
Epcam
Plxna4
Tmigd1
Stk39
Pik3r1
Igfbp3
Anxa5
Dpp4
Lamc2
Kif26a
Dpysl3
Trib1
Mapk15
Dach1
Dapk2
Dock10
Csf1r
Igfbp5
Vegfa
Pitx2
Dysf
Itgav
Angptl3
Adamts1
Apela
Dlc1
Anxa3
Efna1
Apoc3
Scd1
Apoa4
Gls
Fabp5
Ces1f
Pkm
Brca1
Crtap
P4ha1
Me1
Cyp2e1
Gpt2
Odc1
Aldh1l2
Adh7
Adh1
Ephx1
Ldc1
Ugt2b37
Csad
Acmsd
Scd2
Ggt1
Cacnb4
Rgs2
Kcnj8
Dpysl2
Cck
Slc16a6
Slco1b2
Cd36
Slc3a1
Slc1a2
Slc1a4
Slc6a18
Slc5a8
Slc22a2
Mt2
Rab38
Napsa
Nt5e
Bmyc
Unc13b
Cybrd1
Tmem178
Slc4a8
Slc4a11
Wnk2
Atp13a4
Chrna4
Tmc5
Slc23a3
Kcnn1
Tram2
Slc41a2
Slc22a28
Slc17a3
Slc35a3
Col16a1
Itih1
Serpinh1
Elf3
Cpb2
Ptpn14
Fat3
Dusp2
Sh3pxd2a
Pde3b
Tspan12
Klf2
Enpep
Plaur
Igsf3
Frzb
Slitrk6
Mki67
Dzank1
Ly6e
Cdkn1a
Frem2
Cpe
Dusp6
Elmo2
Tubb2b
Tshr
Ptp4a3
Sh3gl2
Kcne4
Fyn
Syne1
Fos
Hsd11b1
Sytl4
Mdfic
Sgcb
Sgcd
Barx2
Adamts15
Krt19
Pgm5
Myom3
Krt8
Adm
Irx3
Npnt
Col4a1
Lrp5
Pou3f3
Cd24a
Gdf11
St14
Tgif1
regulation of locomotion
locomotion
regulation of cell motility
regulation of cell migration
organic acid biosynthesis
organic acid metabolic process
carboxylate metabolism
oxoacid metabolic process
transmembrane transport
amino acid transport
organic acid transport.1
carboxylate transmembrane transport
monocarboxylic acid transport
organic anion transport
carboxylic acid transport
organic acid transport
anatomical morphogenesis
tube development
circulatory system development
muscle cell development
muscle structure development
muscle cell differentiation
striated muscle differentiation
external capsule organization
ECM organization
ECM structure organization
chemical homeostasis
negative regulation of transport
circulatory system process
kidney epithelium development
renal tubule development
epithelium development
animal organ morphogenesis
gland development

Supplementary Fig. 8: **Individual HIF2A-specific upregulated genes driving over-representation in the GO terms.**

Binary heatmap depicting whether individual HIF2A-specific upregulated genes are or are not members of the indicated GO terms. Blue colored tiles represent membership of the gene in the GO term. GO terms and genes are ordered by hierarchical clustering based on the overlapping memberships of GO terms.

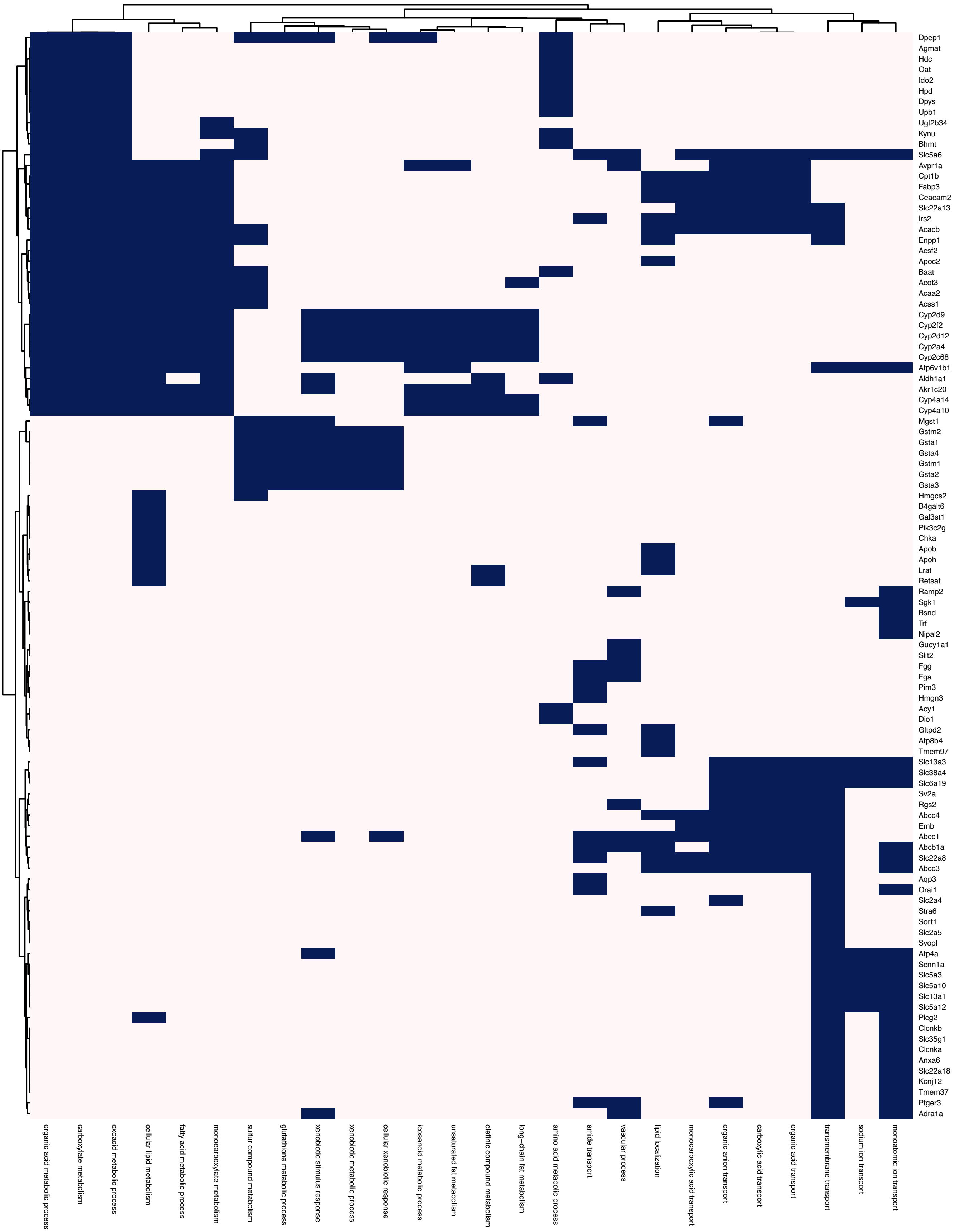
Dpep1
Agmat
Hdc
Oat
Ido2
Hpd
Dpys
Upb1
Ugt2b34
Kynu
Bhmt
Slc5a6
Avpr1a
Cpt1b
Fabp3
Ceacam2
Slc22a13
Irs2
Acacb
Enpp1
Acsf2
Apoc2
Baat
Acot3
Acaa2
Acss1
Cyp2d9
Cyp2f2
Cyp2d12
Cyp2a4
Cyp2c68
Atp6v1b1
Aldh1a1
Akr1c20
Cyp4a14
Cyp4a10
Mgst1
Gstm2
Gsta1
Gsta4
Gstm1
Gsta2
Gsta3
Hmgcs2
B4galt6
Gal3st1
Pik3c2g
Chka
Apob
Apoh
Lrat
Retsat
Ramp2
Sgk1
Bsnd
Trf
Nipal2
Gucy1a1
Slit2
Fgg
Fga
Pim3
Hmgn3
Acy1
Dio1
Gltpd2
Atp8b4
Tmem97
Slc13a3
Slc38a4
Slc6a19
Sv2a
Rgs2
Abcc4
Emb
Abcc1
Abcb1a
Slc22a8
Abcc3
Aqp3
Orai1
Slc2a4
Stra6
Sort1
Slc2a5
Svopl
Atp4a
Scnn1a
Slc5a3
Slc5a10
Slc13a1
Slc5a12
Plcg2
Clcnkb
Slc35g1
Clcnka
Anxa6
Slc22a18
Kcnj12
Tmem37
Ptger3
Adra1a
organic acid metabolic process
carboxylate metabolism
oxoacid metabolic process
cellular lipid metabolism
fatty acid metabolic process
monocarboxylate metabolism
sulfur compound metabolism
glutathione metabolic process
xenobiotic stimulus response
xenobiotic metabolic process
cellular xenobiotic response
icosanoid metabolic process
unsaturated fat metabolism
olefinic compound metabolism
long−chain fat metabolism
amino acid metabolic process
amide transport
vascular process
lipid localization
monocarboxylic acid transport
organic anion transport
carboxylic acid transport
organic acid transport
transmembrane transport
sodium ion transport
monoatomic ion transport

Supplementary Fig. 9: Individual HIF2A-specific downregulated genes driving over-representation in the GO terms.

Binary heatmap depicting whether individual HIF2A-specific downregulated genes are or are not members of the indicated GO terms. Blue colored tiles represent membership of the gene in the GO term. GO terms and genes are ordered by hierarchical clustering based on the overlapping memberships of GO terms.

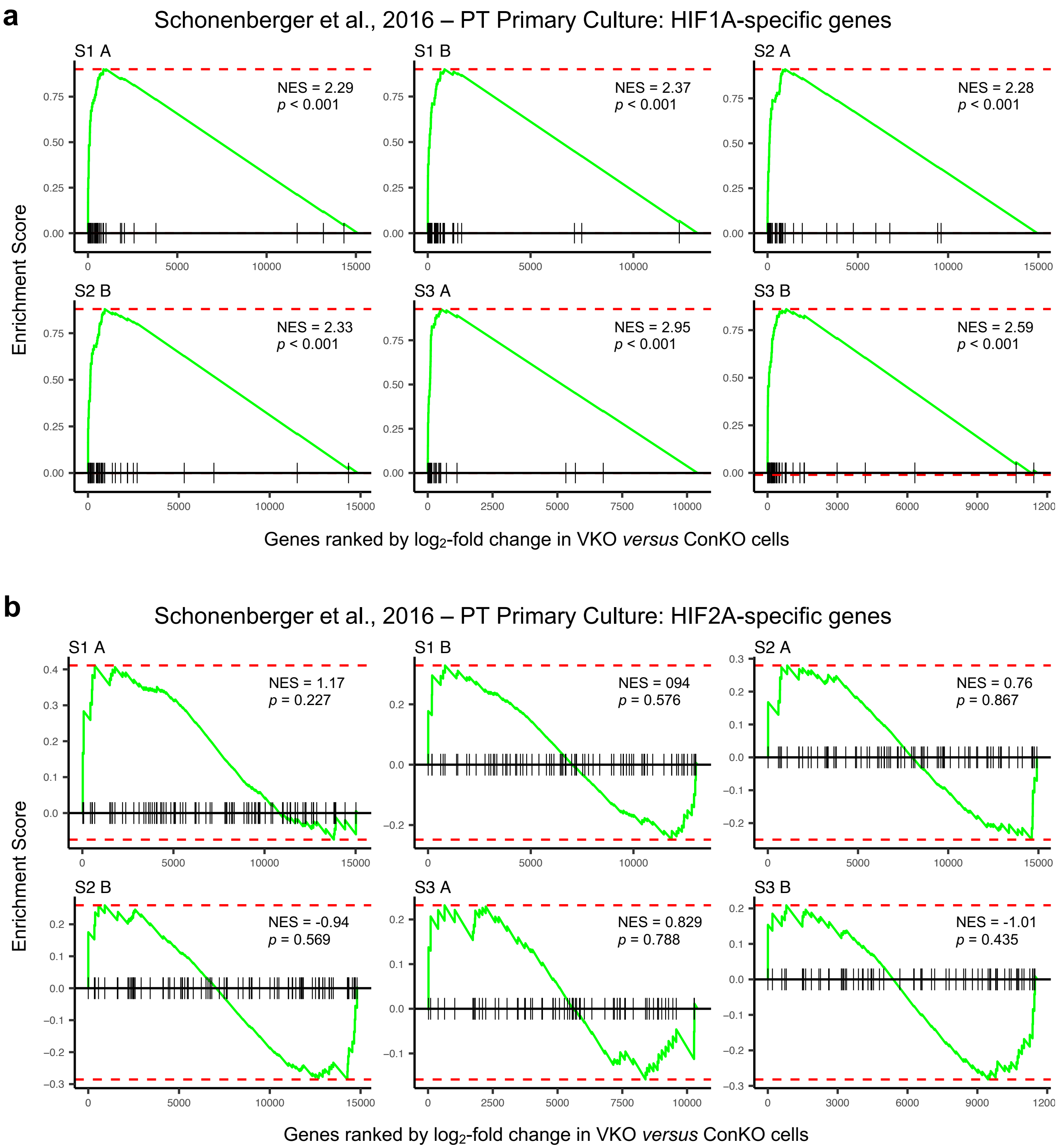
a
Schonenberger et al., 2016 – PT Primary Culture: HIF1A-specific genes
S1 A
NES = 2.29
p < 0.001
S1 B
NES = 2.37
p < 0.001
S2 A
NES = 2.28
p < 0.001
S2 B
NES = 2.33
p < 0.001
S3 A
NES = 2.95
p < 0.001
S3 B
NES = 2.59
p < 0.001
Enrichment Score
Genes ranked by log2-fold change in VKO versus ConKO cells
b
Schonenberger et al., 2016 – PT Primary Culture: HIF2A-specific genes
S1 A
NES = 1.17
p = 0.227
S1 B
NES = 094
p = 0.576
S2 A
NES = 0.76
p = 0.867
S2 B
NES = -0.94
p = 0.569
S3 A
NES = 0.829
p = 0.788
S3 B
NES = -1.01
p = 0.435
Enrichment Score
Genes ranked by log2-fold change in VKO versus ConKO cells

Supplementary Fig. 10: HIF1A-dependent but not HIF2A-dependent gene regulation is conserved *in vitro* and *in vivo* model systems.

Gene-set enrichment analysis (GSEA) on sets of **(a)** HIF1A- and **(b)** HIF2A- dependent genes, as defined in Schonenberger et al., 2016, performed on ranked lists of differentially expressed genes in VKO *versus* ConKO cells of each PT identity. NES – normalized enrichment score.

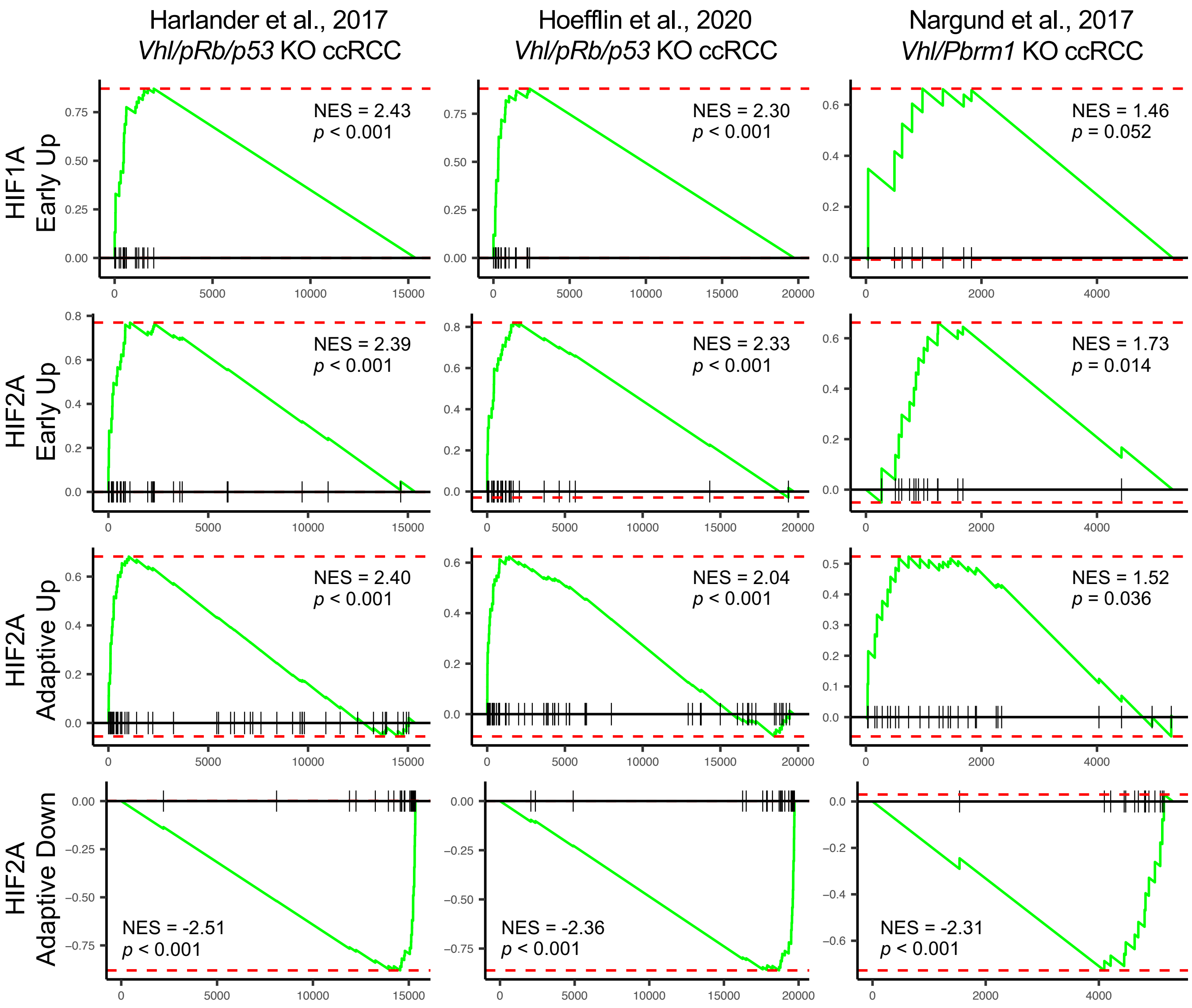

Harlander et al., 2017
Vhl/pRb/p53 KO ccRCC
Hoefflin et al., 2020
Vhl/pRb/p53 KO ccRCC
Nargund et al., 2017
Vhl/Pbrm1 KO ccRCC
HIF1A Early Up
HIF2A Early Up
HIF2A Adaptive Up
HIF2A Adaptive Down
NES = 2.43
p < 0.001
NES = 2.30
p < 0.001
NES = 1.46
p = 0.052
NES = 2.39
p < 0.001
NES = 2.33
p < 0.001
NES = 1.73
p = 0.014
NES = 2.40
p < 0.001
NES = 2.04
p < 0.001
NES = 1.52
p = 0.036
NES = -2.51
p < 0.001
NES = -2.36
p < 0.001
NES = -2.31
p < 0.001

Supplementary Fig. 11: HIFα-dependent 'early' and 'adaptive' transcriptional programs in *Vhl*-null PT cells show significant associations with transcriptional programs in murine ccRCC.

Gene set enrichment analysis (GSEA) of sets of genes upregulated early after *Vhl* inactivation in a manner dependent on HIF1A ('HIF1A Early Up') or HIF2A ('HIF2A Early Up') or regulated specifically over time in *Vhl*-null PT cells in a HIF2A-dependent manner ('HIF2A Adaptive Up' and 'HIF2A Adaptive Down'). The comparisons were performed on three separate publicly-available ranked lists of differentially expressed in murine ccRCC versus normal renal cortex. NES – normalized enrichment score.

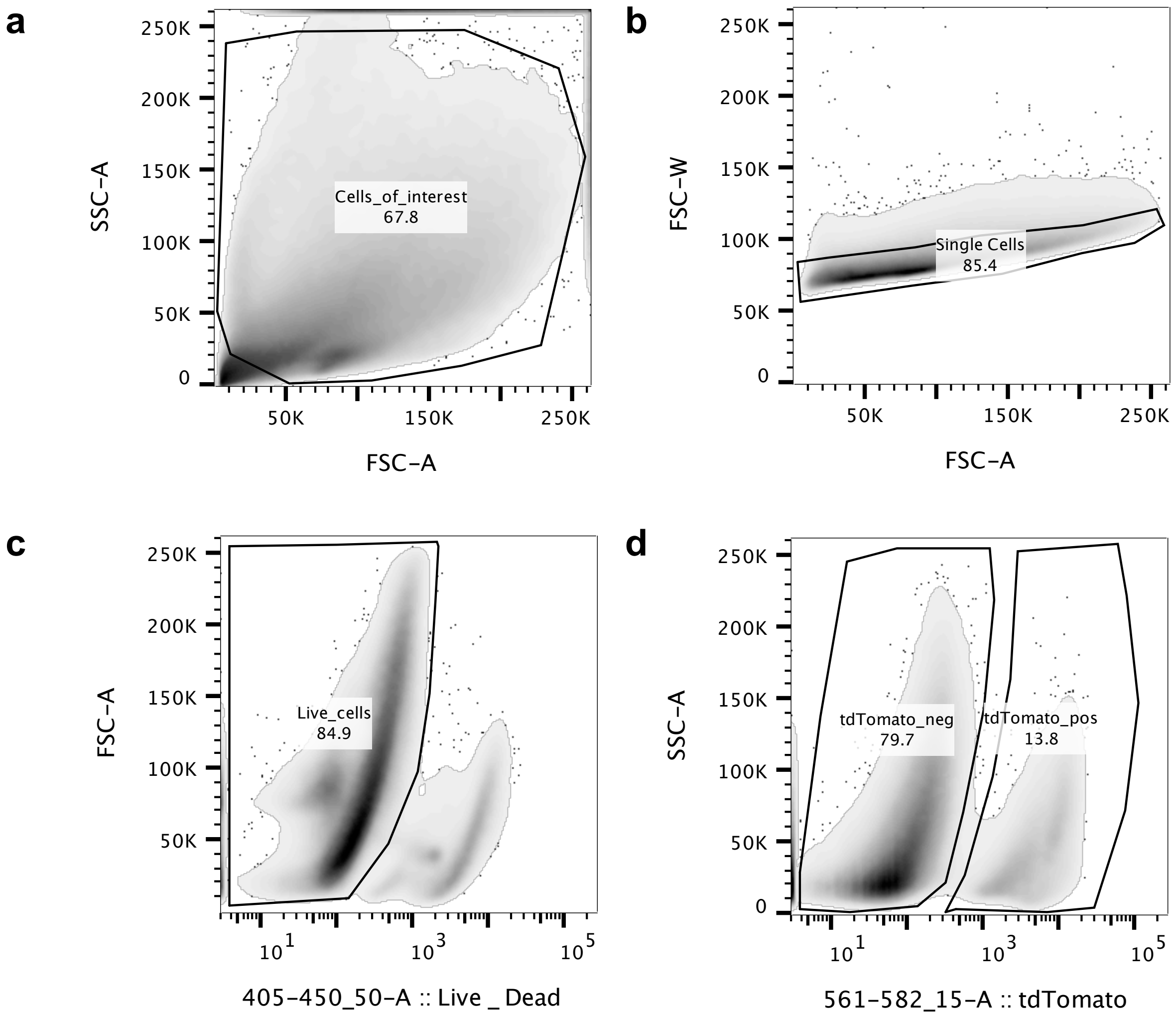
a
b
c
d
SSC-A
FSC-A
Cells_of_interest
67.8
FSC-W
Single Cells
85.4
Live_cells
84.9
405-450_50-A :: Live _ Dead
tdTomato_neg
79.7
tdTomato_pos
13.8
561-582_15-A :: tdTomato
250K
200K
150K
100K
50K
0
50K
150K
250K
10^1
10^3
10^5

Supplementary Fig. 12: Fluorescence-activated cell sorting (FACS) gating strategy used for isolation of tdTomato-positive cells.

Representative density plots depicting the gating strategy to isolate live, single, tdTomato-positive cells from dissociated kidney cell suspension. **(a)** Cells of interest were discerned from debris by gating on Forward Scatter Area (FSC-A) *versus S*ide Scatter area (SSC-A. **(b)** Single cells were discerned from doublets and aggregates by gating the cells of interest on FSC-A vs. Forward Scatter Width (FSC-W). **(c)** Live cells were identified as single cells not stained positive by DAPI, which was detected using a 405-450_50 band/pass filter. **(d)** tdTomato-positive cells were isolated from the live, single cell population based on the positive signal detected with the 561-582_15 band/pass filter. The percentage of cells included in each gate is indicated.
