# Supplementary material for: HIFα isoform specific activities drive cell-type specificity of VHL-associated oncogenesis
Source: Nat Commun. 2025 Oct 16;16:9185. doi: 10.1038/s41467-025-64214-3 (PMC12533196; doi:10.1038/s41467-025-64214-3)
Supplement: Supplementary file 2 — Description of Additional Supplementary Files [file 41467_2025_64214_MOESM2_ESM.pdf]

Description of Additional Supplementary Files

Supplementary Data 1: Lists of genes that are members of PT Module A and Module B.

Supplementary Data 2: Pseudo-bulked differential gene expression analysis performed separately in cells of each PT identity, indicating Vhl- and HIFα-dependent effects.

Supplementary Data 3: Lists of renal cell-type specific marker genes used to assign cell type in scRNA-seq analysis
