# Supplementary material for: HIFα isoform specific activities drive cell-type specificity of VHL-associated oncogenesis
Source: Nat Commun. 2025 Oct 16;16:9185. doi: 10.1038/s41467-025-64214-3 (PMC12533196; doi:10.1038/s41467-025-64214-3)
Supplement: Supplementary file 6 — Reporting Summary [file 41467_2025_64214_MOESM6_ESM.pdf]

nature portfolio

Corresponding author(s): Peter J Ratcliffe, Samvid Kurlekar

Last updated by author(s): Aug 15, 2025

Reporting Summary

Nature Portfolio wishes to improve the reproducibility of the work that we publish. This form provides structure for consistency and transparency in reporting. For further information on Nature Portfolio policies, see our Editorial Policies and the Editorial Policy Checklist.

Statistics

For all statistical analyses, confirm that the following items are present in the figure legend, table legend, main text, or Methods section.

n/a	Confirmed	
☐	☒	The exact sample size (*n*) for each experimental group/condition, given as a discrete number and unit of measurement
☐	☒	A statement on whether measurements were taken from distinct samples or whether the same sample was measured repeatedly
☐	☒	The statistical test(s) used AND whether they are one- or two-sided<br>*Only common tests should be described solely by name; describe more complex techniques in the Methods section.*
☐	☒	A description of all covariates tested
☐	☒	A description of any assumptions or corrections, such as tests of normality and adjustment for multiple comparisons
☐	☒	A full description of the statistical parameters including central tendency (e.g. means) or other basic estimates (e.g. regression coefficient) AND variation (e.g. standard deviation) or associated estimates of uncertainty (e.g. confidence intervals)
☐	☒	For null hypothesis testing, the test statistic (e.g. *F*, *t*, *r*) with confidence intervals, effect sizes, degrees of freedom and *P* value noted<br>*Give P values as exact values whenever suitable.*
☒	☐	For Bayesian analysis, information on the choice of priors and Markov chain Monte Carlo settings
☒	☐	For hierarchical and complex designs, identification of the appropriate level for tests and full reporting of outcomes
☒	☐	Estimates of effect sizes (e.g. Cohen's *d*, Pearson's *r*), indicating how they were calculated

Our web collection on statistics for biologists contains articles on many of the points above.

Software and code

Policy information about availability of computer code

Data collection	No software was used for data collection.
Data analysis	The following softwares were used to perform data analysis: pheatmap (v1.0.12), ggvenn (v0.1.10), ggplot2 (v3.5.1), R (v4.3.2 and v4.4.1), GraphPad Prism 10, clusterProfiler (v4.12.6),DESeq2 (v1.44.0), UCell (v2.8.0),Seurat (v4.0.3 and v5.1.0), CellRanger (v6.1.1), HALO Image Analysis Software (v3.5 and v3.6)

For manuscripts utilizing custom algorithms or software that are central to the research but not yet described in published literature, software must be made available to editors and reviewers. We strongly encourage code deposition in a community repository (e.g. GitHub). See the Nature Portfolio guidelines for submitting code & software for further information.

Data

Policy information about availability of data

All manuscripts must include a data availability statement. This statement should provide the following information, where applicable:

- Accession codes, unique identifiers, or web links for publicly available datasets
- A description of any restrictions on data availability
- For clinical datasets or third party data, please ensure that the statement adheres to our policy

The scRNA-seq data for Vhl-null cells (ConKO and VKO) harvested early and late after recombination data used in this study are available in the Gene Expression Omnibus (GEO) database under accession code GSE253168 [https://www.ncbi.nlm.nih.gov/geo/query/acc.cgi?acc=GSE253168]. The scRNA-seq data generated in

this study for VHKO, VEKO, and VHEKO mice harvested late after recombination are available in the GEO database under accession code GSE282887 [https://www.ncbi.nlm.nih.gov/geo/query/acc.cgi?acc=GSE282887]. Source data for IHC quantification analyses are provided with this paper.

Research involving human participants, their data, or biological material

Policy information about studies with human participants or human data. See also policy information about sex, gender (identity/presentation), and sexual orientation and race, ethnicity and racism.

Reporting on sex and gender	*Use the terms sex (biological attribute) and gender (shaped by social and cultural circumstances) carefully in order to avoid confusing both terms. Indicate if findings apply to only one sex or gender; describe whether sex and gender were considered in study design; whether sex and/or gender was determined based on self-reporting or assigned and methods used. Provide in the source data disaggregated sex and gender data, where this information has been collected, and if consent has been obtained for sharing of individual-level data; provide overall numbers in this Reporting Summary. Please state if this information has not been collected. Report sex- and gender-based analyses where performed, justify reasons for lack of sex- and gender-based analysis.*
Reporting on race, ethnicity, or other socially relevant groupings	*Please specify the socially constructed or socially relevant categorization variable(s) used in your manuscript and explain why they were used. Please note that such variables should not be used as proxies for other socially constructed/relevant variables (for example, race or ethnicity should not be used as a proxy for socioeconomic status). Provide clear definitions of the relevant terms used, how they were provided (by the participants/respondents, the researchers, or third parties), and the method(s) used to classify people into the different categories (e.g. self-report, census or administrative data, social media data, etc.) Please provide details about how you controlled for confounding variables in your analyses.*
Population characteristics	*Describe the covariate-relevant population characteristics of the human research participants (e.g. age, genotypic information, past and current diagnosis and treatment categories). If you filled out the behavioural & social sciences study design questions and have nothing to add here, write "See above."*
Recruitment	*Describe how participants were recruited. Outline any potential self-selection bias or other biases that may be present and how these are likely to impact results.*
Ethics oversight	*Identify the organization(s) that approved the study protocol.*

Note that full information on the approval of the study protocol must also be provided in the manuscript.

Field-specific reporting

Please select the one below that is the best fit for your research. If you are not sure, read the appropriate sections before making your selection.

☒ Life sciences ☐ Behavioural & social sciences ☐ Ecological, evolutionary & environmental sciences

For a reference copy of the document with all sections, see nature.com/documents/nr-reporting-summary-flat.pdf

Life sciences study design

All studies must disclose on these points even when the disclosure is negative.

Sample size	Sample sizes were not determined by statistical testing. At least n = 4 mice were used for each genotype and timepoint for each experiment, including single-cell RNA sequencing analysis. The precise number of mice used in each experiment has been described in the figures or figure legends.
Data exclusions	No data was excluded.
Replication	For histological analysis and quantitative analysis of cell survival and proliferation, experiments were performed at multiple instances over two years. At least n = 4 and up to n = 17 mice were analyzed for each genotype and timepoint. Mice of multiple genotypes and timepoints were included in every round of immunohistochemical staining to avoid batch and handling effects from confounding biological effects. Single-cell RNA sequencing analysis was performed on n = 4 mice of each genotype and timepoint, with multiple genotypes and timepoints included in each round of tissue dissociation, single-cell GEMM preparation, and sequencing. Reproducibility was assessed by comparing the global transcriptomic profiles of cells of the same genotype and timepoint but derived from different mice processed on different days (see Supplementary Fig. 4)
Randomization	Formal randomization was not used. When possible, experimental mice belonging to the same litter were split between the early and late timepoints.
Blinding	Blinding to the genotype or timepoint was not possible as the investigators who bred and genotyped the animals were the same as those who performed the interventions, experiments and analysis. However, morphometry was performed using the same pre-specified parameters for all genotypes and timepoints.

Reporting for specific materials, systems and methods

We require information from authors about some types of materials, experimental systems and methods used in many studies. Here, indicate whether each material, system or method listed is relevant to your study. If you are not sure if a list item applies to your research, read the appropriate section before selecting a response.

Materials & experimental systems

n/a	Involved in the study
☐	☒ Antibodies
☒	☐ Eukaryotic cell lines
☒	☐ Palaeontology and archaeology
☐	☒ Animals and other organisms
☒	☐ Clinical data
☒	☐ Dual use research of concern
☒	☐ Plants

Methods

n/a	Involved in the study
☒	☐ ChIP-seq
☐	☒ Flow cytometry
☒	☐ MRI-based neuroimaging

Antibodies

Antibodies used	tdTomato antibody (Rockland 600-401-379, RRID:AB_2209751) , Ki67 biotin-conjugated antibody (Life Technologies 13569882, RRID: AB_2572794)
Validation	Both antibodies are of commerical origin and have been validated by the manufacturer

Animals and other research organisms

Policy information about studies involving animals; ARRIVE guidelines recommended for reporting animal research, and Sex and Gender in Research

Laboratory animals	B6.Vhltm1.1b(tdTomato)Pjr (Vhlpjr.fl) mice were commissioned from Ozgene, Australia and generated using goGermline technology. Vhltm1jae mice (Vhljae.fl) (RRID: IMSR_JAX:012933) were crossed with Tg(Pgk1-cre)1Lni (Pgk1-Cre) (RRID: IMSR_JAX:020811) to generate a constitutively inactivated Vhltm1jae allele (Vhljae.KO). Tg(Pax8-cre/ERT2)CAmat (Pax8-CreERT2) (RRID: IMSR_HAR:9175) mice were obtained via EMMA. Mice carrying conditional alleles for Hif1a (Hif1atm3Rsjo; RRID: MGI:6863863; termed Hif1afl) and Epas1 (Epas1tm1.1Mcs; RRID:MGI:3710345; termed Epas1fl) were gifts from Randall Johnson and M Celeste Simon respectively. These lines were generated on a B6.129 mixed background but were inter-crossed with C57B6/J animals for at least five generations prior to starting experiments. Mice of both sexes were administered tamoxifen (2 mg dose daily for five consecutive days) when they were >20 g in body weight, which occurred when male mice were ~6 weeks old and female mice were ~10 weeks old. Mice were harvested either 1-3 weeks (early) or 4-12 months (late) after recombination was induced.
Wild animals	Wild animals were not used for this study
Reporting on sex	Mice of both sexes were used. Sex was considered as a biological replicate for gene expression analysis as gene expression in the renal proximal tubule is sexually dimorphic. The conclusions of the study apply to both sexes.
Field-collected samples	Samples were not collected in the field
Ethics oversight	All experimental procedures were conducted following approval by the Medical Science Ethical Review Committee of the University of Oxford and authorized under UK Home Office regulations of Animals (Scientific Procedures) Act 1986.

Note that full information on the approval of the study protocol must also be provided in the manuscript.

Plants

Seed stocks	*Report on the source of all seed stocks or other plant material used. If applicable, state the seed stock centre and catalogue number. If plant specimens were collected from the field, describe the collection location, date and sampling procedures.*
Novel plant genotypes	*Describe the methods by which all novel plant genotypes were produced. This includes those generated by transgenic approaches, gene editing, chemical/radiation-based mutagenesis and hybridization. For transgenic lines, describe the transformation method, the number of independent lines analyzed and the generation upon which experiments were performed. For gene-edited lines, describe the editor used, the endogenous sequence targeted for editing, the targeting guide RNA sequence (if applicable) and how the editor was applied.*
Authentication	*Describe any authentication procedures for each seed stock used or novel genotype generated. Describe any experiments used to assess the effect of a mutation and, where applicable, how potential secondary effects (e.g. second site T-DNA insertions, mosiacism, off-target gene editing) were examined.*

Flow Cytometry

Plots

Confirm that:

☒ The axis labels state the marker and fluorochrome used (e.g. CD4-FITC).

☒ The axis scales are clearly visible. Include numbers along axes only for bottom left plot of group (a 'group' is an analysis of identical markers).

☒ All plots are contour plots with outliers or pseudocolor plots.

☒ A numerical value for number of cells or percentage (with statistics) is provided.

Methodology

Sample preparation	Kidneys were bisected, the renal capsule removed, and then macerated for up to 7 min on a bed of ice. Macerated kidneys were then subjected to single-cell dissociation using the Multi-Tissue Dissociation Kit 2 (Miltenyi 130-110-203). Briefly, macerated tissues were suspended in 1.45 ml Buffer X, 30 l of Enzyme D, 15 l of Enzyme P, 15 l of Buffer Y, and 6 l of Enzyme A, all prepared according to the manufacturer's instructions. The suspension was then incubated under water at 37° C for 30 min in a shaking incubator set to 150 rpm. The procedure was stopped by the addition of 150 l fetal bovine serum (FBS; Sigma F7524) and resuspension in 9 ml of RPMI-1640 medium (Merck R0883). The digest was filtered through a 40 m cell strainer to remove undigested tissue and the filtrate was centrifuged (300g for 10 min at 4°C). Erythrocytes were eliminated by resuspending dissociated cells in 3 ml of 1x RBC Lysis Buffer (Miltenyi 130-094-183) prepared in deionized water and incubating for 2 min at RT. Cells were centrifuged (300g for 5 min at 4°C) and resuspended in ice-cold D-PBS before being counted on a Thermo Scientific Countess II machine for total yield and viability.
Instrument	Cells were sorted using a BD Aria Fusion Cell Sorter
Software	Data was analyzed using FlowJo 10.10.0
Cell population abundance	Live, single, tdTomato-positive cells were between 4-12% of the total dissociated cell population.
Gating strategy	Cells of interest were selected over cellular debris using a FSC-A vs SSC-A gate. Single cells were selected on the basis of a FSC-A vs FSC-W gate. Live cells were selected as those not stained by DAPI detected with a 405 laser excitation and a 450/40 band pass filter. Within live cells, tdTomato-positive cells were selected as those positively detected with a 561 nm laser excitation and a 582/15 band pass filter.

☒ Tick this box to confirm that a figure exemplifying the gating strategy is provided in the Supplementary Information.
